# Prior Pronunciation Knowledge Bootstraps Word Learning

**DOI:** 10.3389/fcomm.2018.00001

**Published:** 2018-02-05

**Authors:** Khia Anne Johnson, Gloria Madeleine Mellesmoen, Roger Yu-Hsiang Lo, Bryan Gick

**Affiliations:** 1 Department of Linguistics, University of British Columbia, Vancouver, BC, Canada; 2 Haskins Laboratories, New Haven CT, USA

**Keywords:** L2 acquisition, bootstrapping, speech perception, speech production, word learning, pronunciation training

## Abstract

Learners often struggle with L2 sounds, yet little is known about the role of prior pronunciation knowledge and explicit articulatory training in language acquisition. This study asks if existing pronunciation knowledge can bootstrap word learning, and whether short-term audiovisual articulatory training for tongue position with and without a production component has an effect on lexical retention. Participants were trained and tested on stimuli with perceptually salient segments that are challenging to produce. Results indicate that pronunciation knowledge plays an important role in word learning. While much about the extent and shape of this role remains unclear, this study sheds light in three main areas. First, prior pronunciation knowledge leads to increased accuracy in word learning, as all groups trended toward lower accuracy on pseudowords with two novel segments, when compared with those with one or none. Second, all training and control conditions followed similar patterns, with training neither aiding nor inhibiting retention; this is a noteworthy result as previous work has found that the inclusion of production in training leads to decreased performance when testing for retention. Finally, higher production accuracy during practice led to higher retention after the word-learning task, indicating that individual differences and successful training are potentially important indicators of retention. This study provides support for the claim that pronunciation matters in L2 word learning.

## 1. INTRODUCTION

One of the first challenges in learning a second language (L2) is producing and perceiving unfamiliar sounds. This is described in speech learning models where the perception of non-native phonemes is influenced by first language (L1) phonetic categories (e.g., Best, 1995; Flege, 1995; Kuhl et al., 2008). Despite extensive research regarding how learners acquire or fail to acquire L2 sounds, relatively few studies have focused on the role that existing pronunciation knowledge plays in acquiring higher level elements of language, such as particular morphemes or words. This study asks whether existing pronunciation knowledge bootstraps word learning. Related to this, we also ask whether short-term pronunciation training affects retention. Considering the complicated interplay between perception and production in speech learning, training with and without a production component is considered.

As a phenomenon in language acquisition, bootstrapping has been studied much more thoroughly from the perspective of language development in children. There is strong empirical evidence that infants benefit from bootstrapping, using existing knowledge of phonetics and phonology to further develop other aspects of acquisition such as word learning (Werker and Yeung, 2005), syntax, and prosody (Morgan and Demuth, 1996). Bootstrapping has not been studied as thoroughly in L2 acquisition, although existing literature does suggest a connection between pronunciation knowledge and word learning. For example, the ability to discriminate between non-native sounds predicts word-learning accuracy (Silbert et al., 2015), and perceptual training also leads to improved discrimination ability and word learning (Perfors and Dunbar, 2010). L2 learners are also sensitive to information concerning where the sound surfaces, and this word-level information can influence category development when trained alongside semantic content in a word-learning task (Hayes-Harb, 2007), but also in the absence of semantic content (Feldman et al., 2013). While these studies suggest a close tie between pronunciation and word learning, their focus has been on perceptually challenging contrasts and perceptual training and learning, without focusing on how these interact with existing and trained pronunciation knowledge.

There is still much more to be understood about the relationship between production and perception in L2 acquisition. While prior work on L1 production and perception suggests a cooperative relationship throughout development (Kuhl et al., 2008), studies examining L2 production and perception paint a decidedly less cohesive picture. Positive correlations between production and perception have been found for English speakers learning Mandarin lexical tones in some cases (Wang et al., 2003), but not in others (Bent, 2005). Further, a number of studies demonstrate that including a production component in training can disrupt perceptual learning. For example, Baese-Berk and Samuel (2016) used an ABX training paradigm over 2 days, where participants received feedback, and half were asked to repeat the last token aloud. When tested with the same ABX paradigm without feedback, participants who produced the items aloud performed worse. As a second example, using different methods, Morrison and Hudson Kam (2009) trained participants during a single session to passively associate words with images, with additional training conditions involving different types of practice. Again, they found that practicing aloud inhibited retention. Regardless of whether production plays an inhibitory role or the two modalities work in concert, previous studies have demonstrated that there is substantial individual variability (e.g., Bradlow et al., 1997, 1999; Wang et al., 2003).

Given the complicated interaction between perception and production, it is important to consider both in articulatory training. This study addresses this in two ways, encapsulated within each of the research questions. The first addresses the role that prior pronunciation knowledge plays in L2 word learning, while the second looks at the role of short-term training, as the goal of this type of research is to discover how to best assist learners in acquiring an L2. We approach these questions by testing participants on stimuli that are perceptually salient, yet challenging to produce. This will help disambiguate the relative roles of perception and production.

We hypothesize that existing pronunciation knowledge will bootstrap word learning, considering the preliminary evidence that word-learning accuracy decreases when words contain complex consonant clusters (Morrison and Hudson Kam, 2009). While this provides strong motivation for our hypothesis, it remains unclear from previous literature whether the decrease in accuracy is due to the individual segments themselves or their combination. In addition, none of the novel segments and clusters in Morrison and Hudson Kam (2009) included challenging vowels. With respect to training, we predict increased accuracy on pseudowords with novel segments for participants with pronunciation training, compared with the group with no training. This follows from the finding that pronunciation training can lead to improvements in word learning (Perfors and Dunbar, 2010). For the training with production group, we entertain two competing hypotheses. On one hand, there is evidence that production inhibits perceptual learning on a number of levels (Morrison and Hudson Kam, 2009; Baese-Berk and Samuel, 2016). On the other hand, others have found that the inclusion of production during a picture association training task over five sessions improves accuracy when listeners are tasked with recognizing the word in noise, but that it simultaneously impairs lexical engagement (Leach and Samuel, 2007). The investigation of our two research questions will shed light on the importance of pronunciation knowledge, and the role that training can play for L2 learners.

## 2. MATERIALS AND METHODS

### 2.1. Participants

The participants were 61 undergraduate students (39 female, 21 male, and 1 non-binary) at the University of British Columbia (UBC) enrolled in one or more lower level linguistics courses,^1^ between the ages of 18 and 27, with a mean age of 19.61. The students received course credit or $10 CAD in exchange for their participation. All participants signed a consent form before the start of the experiment and were informed that they could change their mind at any point during the experiment and would still be compensated for their time. A total of 124 participants received course credit for this experiment, but 63 participants were excluded at the point of analysis. This is a fairly normal rate of exclusion at UBC, due to a high degree of linguistic diversity in the student population. The exclusion criteria were set before the experiment and included conflicting language backgrounds,^2^ producing the pseudo words when they were not prompted to do so, advanced phonetics coursework (see text footnote 1), or self-reported speech and hearing disorders. Data from six participants were excluded due to missing audio files or other experimenter error. Participants were assigned randomly to one of two experimental conditions or the Control group (n = 21), which received no training. The two experimental conditions were Audiovisual Perception (n = 20) and Audiovisual Production (n = 20) training.

### 2.2. Stimuli

The stimuli for the word-learning task were pseudowords, which had a CVCV shape. There were a total of 64 lexical items: 16 were of Familiar-Only type, composed of only segments present in English ([f], [ɡ], [l], [m], [t], [i], [ ɛ], [ɑ], or [u]), 16 were of Novel-V type, containing one novel vowel ([ø] or [ɯ]) in the first vowel position, 16 were of Novel-C type, containing one novel consonant ([k͡| ] or [χ]) in C2, and 16 were of Novel-CV type, containing one novel vowel and consonant in V1 and C2, respectively (see Appendix). The novel vowels [ø] and [ɯ] and the consonants [k͡| ], and [χ] were chosen because they were not present in the native languages of the anticipated participants^3^ and were therefore deemed comparatively difficult to articulate, yet still perceptually salient. It is worth noting, however, that the novel consonants are more salient than the vowels, as they are considerably different from the consonants present in English, while the vowels are situated relatively closer to other phonemes in English^4^ because of the large and complex vowel system of English (Maddieson, 1984). Each lexical item was produced by a single trained phonetician, rather than synthesized, to maximize the naturalness and retain consistency in pronunciation across all stimuli.

**Figure 1.**
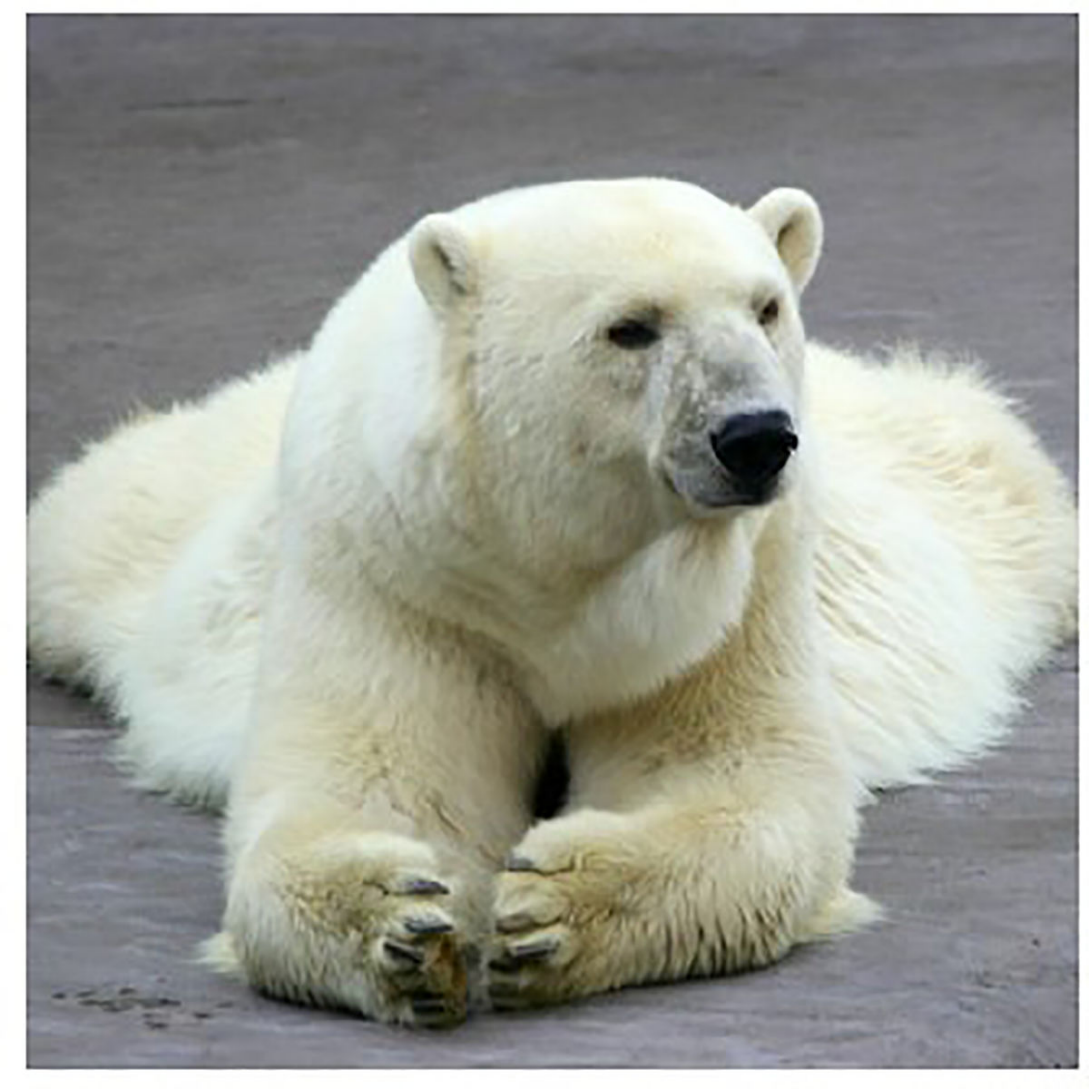
This stock image of a polar bear was paired with the audio for [luχɑ].

Each lexical item was paired with a stock photo of an animal taken from Morguefile (https://morguefile.com/), an open source image site. This is exemplified in Figure 1. These images all belonged to the same semantic category to minimize interference related to the meaning attributed to each pseudoword. We also ensured that the pseudowords did not share more than one segment with the word corresponding to the same animal in English, Mandarin, and Cantonese, which were the expected native languages of the participants, to mitigate the possibility that they might be perceived as cognates. Given the requirement for the pictures to fall within the same semantic category and not share segments between the three languages, we were not able to control for word frequency.

### 2.3. Training Materials

**Figure 2.**
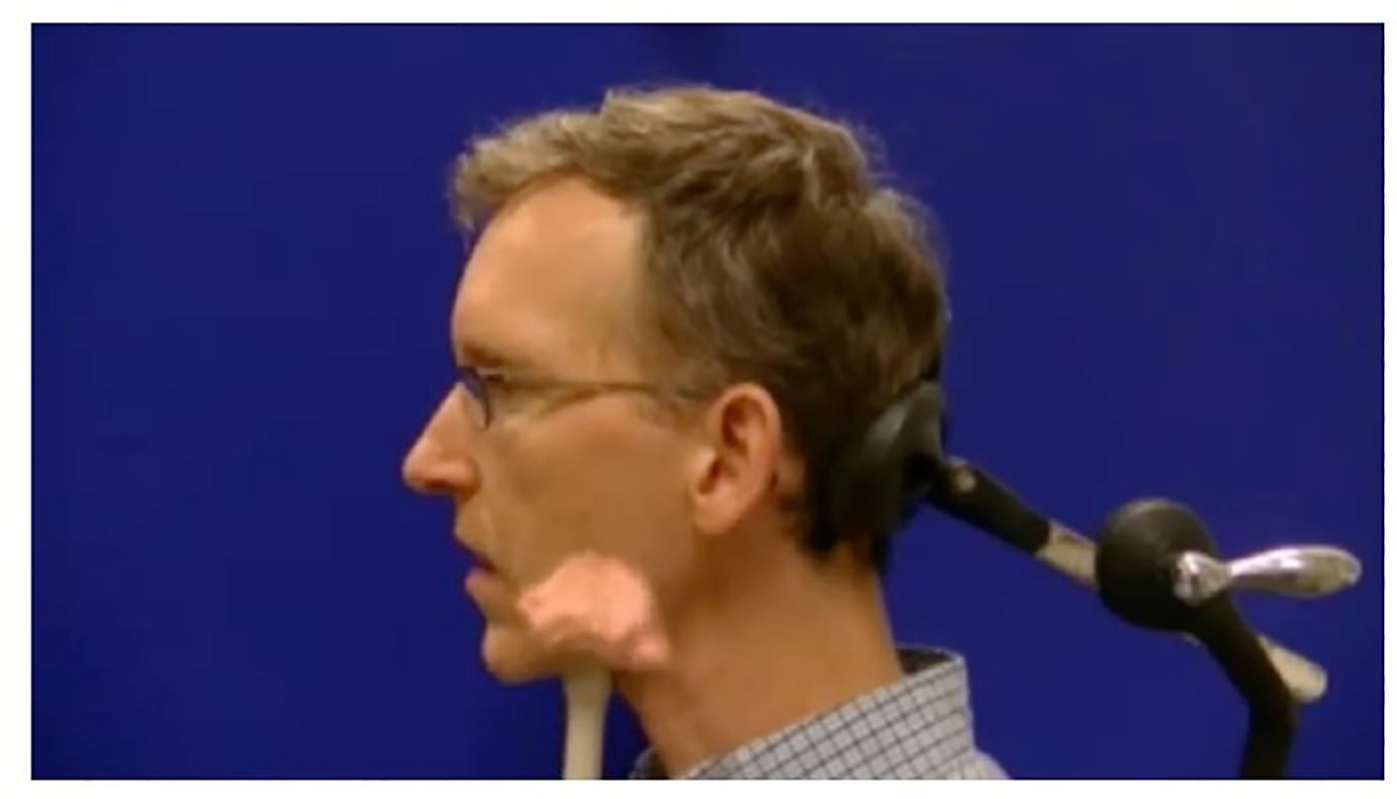
A still image extracted from the ultrasound overlay video for [χ]. Written informed consent was obtained from the individual for the publication of this image.

Participants in the Audiovisual Perception and Audiovisual Production conditions were trained with nearly identical materials. The only difference between the groups was that the Audiovisual Production was prompted to produce the sounds during the training block of the experiment, and provided with an appropriate amount of time to do so. The training materials for each sound ([χ], [k͡| ], [ø], and [ɯ]) consisted of a text description, an ultrasound overlay video depicting tongue movement, and three audio examples (CV for the vowels and VCV for consonants). The text and audio descriptions for both training groups included orthographic representations of the novel sounds, to help participants identify what they needed to learn (Bassetti et al., 2015). The ultrasound overlay videos depicted a speaker producing a sound, with ultrasound video of their tongue superimposed on a midsagittal view of the head, as in Figure 2. The videos used here were developed as a part of the UBC eNunciate project (eNunciate!, 2017). To ensure the clarity of the ultrasound overlay videos, participants first watched a video outlining how to interpret them before the training blocks for specific sounds. All training and testing materials were shown to the participants on the same desktop computer.

Participants were trained on the sounds in a random order. The sequence was fixed within the training blocks for each sound. Training was self-paced, and participants were presented with material in the following order: text, audio, video, audio, video, and finally, audio again. Repetition of the individual materials was used to build up learner understanding of the articulations, without moving too quickly from one sound to the next. Participants in the Audiovisual Production condition were prompted to repeat after each audio example, resulting in nine repetitions per word (3 repetitions per word in each audio section × 3 audio sections in the block). Training lasted approximately 15–20 min depending on the condition and participant self-pacing.

### 2.4. Procedure

Participants in all conditions were told that they would be learning words in a fictional language. The experiment had three sections: training, learning, and testing. During the training phase, the participants assigned to the control condition read a fictional article about the language, without mention of the sound system. This was intended to mentally prepare them to learn a new language, without providing any phonetic information. Those in the Audiovisual Perception condition received pronunciation training, as specified in Section 2.3. Further, participants in this group were instructed not to produce any sounds during the training phase. Those in the Audiovisual Production training condition were presented with identical training materials to the perception condition, with the key difference being that they were prompted to repeat after each audio example, as specified in Section 2.3.

During the word-learning phase, participants from the three conditions were exposed to a word-learning task, modeled after (Morrison and Hudson Kam, 2009). They saw a stock photo of an animal and heard a corresponding lexical item. Care was taken to ensure that the animals in the photos were different enough to prevent confusion within a particular species. For each word-image pairing, participants saw the image for 3,500 ms and heard the word one time, 500 ms after the image appeared. Five hundred milliseconds of silence separated the presentation of each image. Participants in all conditions were instructed not to repeat the words out loud during the word-learning phase. There were a total of four blocks, with each word-image pair presented once in random order within the block. The word-learning phase lasted approximately 18 minutes.

In the subsequent testing phase, participants completed a forced-choice word recognition task with three options. In each test set, participants saw three animal images they had been exposed to during the word-learning phase, each paired with a number (1, 2, or 3). When they were ready, they pressed the spacebar to play the word and provided their answer by pressing the corresponding number key. While participants were only allowed to listen to each word once, self-pacing ensured that they were prepared to listen to the next item. Before the test, participants first completed a short practice test with familiar words (English fruit names) to ensure that they were comfortable with the task. Following the practice, participants were tested on all 64 words, in random order. Afterward, the participants were asked to fill out a language background questionnaire, which marked the end of the experiment.

Participants were seated individually in a sound attenuated booth for the duration of the experiment. After starting the experiment, the experimenter left the booth. Audio for all participants was recorded using Audacity 2.1.2 and a Samson C03U USB-condenser microphone through the duration of the experiment. Recording provided a mechanism to ensure that instructions were followed with respect to production. Given the complicated role that production plays in phonetic and lexical learning, recording the participants’ productions in the Audiovisual Production group also enabled the examination of how accuracy in production interacts with word learning.

## 3. RESULTS

### 3.1. Word-Learning Retention Results

The data were analyzed using a series of logistic mixed effects models fit with retention test accuracy as the dependent variable. Fixed effects included training condition (Control, Audiovisual Perception, or Audiovisual Production) and stimulus type (Familiar-Only, Novel-C, Novel-V, or Novel-CV), as well as their interaction. Both training condition and stimulus type were coded using treatment (dummy) coding, with different combinations of training condition and stimulus type comprising the reference levels. The random effects structure was as maximally specified as possible, with Subject and Stimulus as random intercepts, by-Subject as a random slope for stimulus type, and by-Stimulus as a random slope for training condition. In total, 12 logistic mixed effects models were fit (3 training conditions x 4 stimulus types).

First, we compared participant performance within each training condition; the results are summarized in Table 1. In the Control condition, participants generally achieved higher accuracy on stimuli with one novel consonant (Novel-C) than those with two novel sounds (Novel-CV) (β = 0.43, SE = 0.23, z = 1.86, p = 0.063), although the difference does not reach the threshold for significance of p = 0.05. In the Audiovisual Perception condition, stimulus type was not a significant predictor of accuracy on the retention task. In the Audiovisual Production condition, participants performed significantly better on stimuli without novel sounds (Familiar-Only) or with just one novel consonant (Novel-C), as compared with two novel sounds (Novel-CV) (Familiar-Only vs. Novel-CV: β = 0.45, SE = 0.23, z = 1.97, p = 0.048; Novel-C vs. Novel-CV: β = 0.47, SE = 0.21, z = 2.22, p = 0.026). Results reported in this paragraph are only those reaching or nearly reaching significance. Refer to Table 1 for all other results.

**Figure 3.**
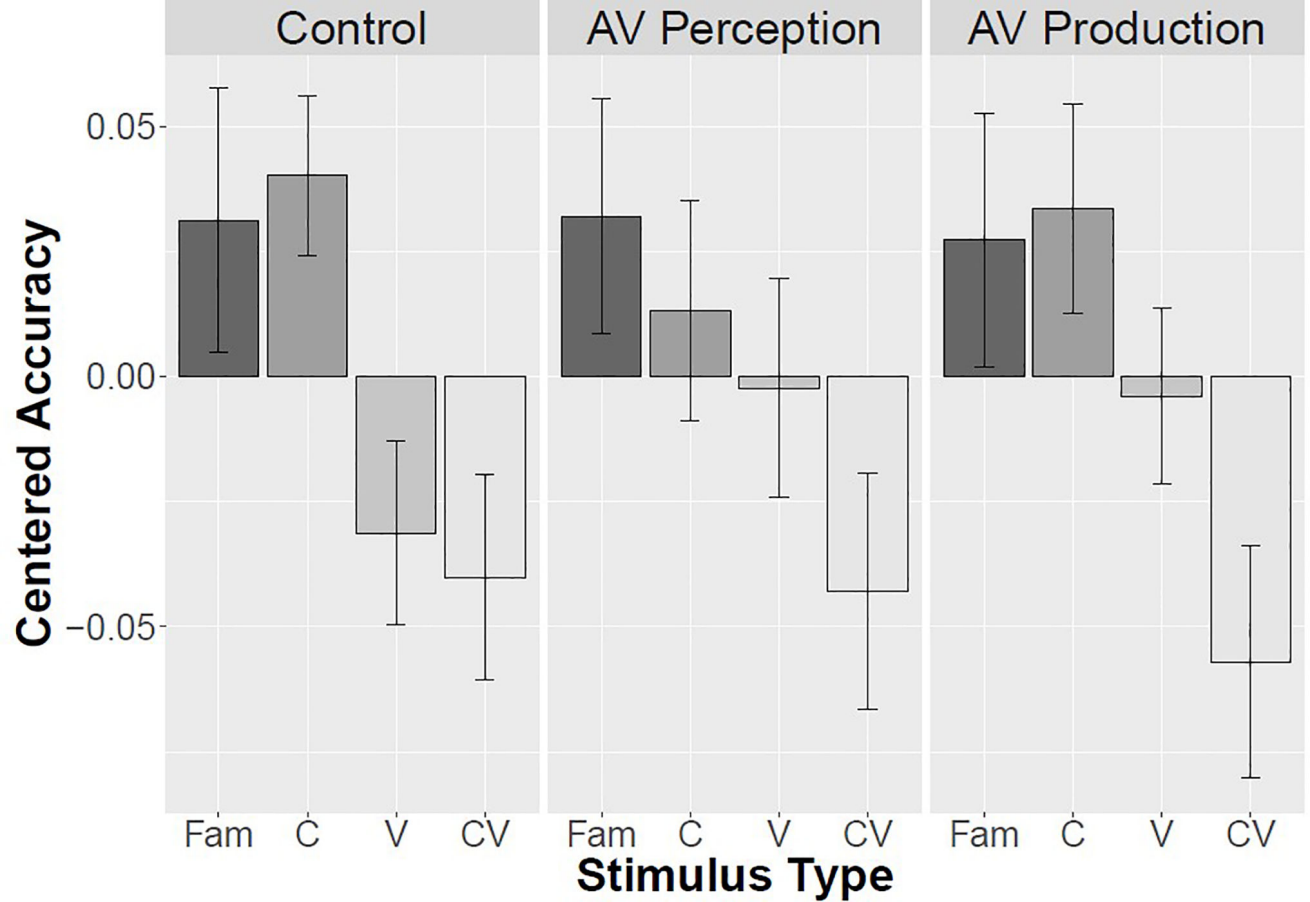
Mean centered accuracy by stimulus type across training conditions. The vertical bars indicate standard error of the mean.

**Figure 4.**
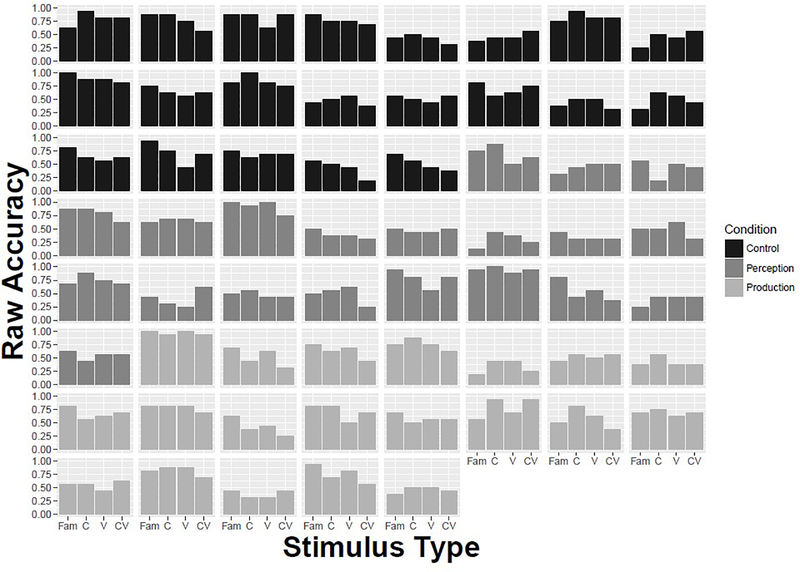
Raw mean accuracy by stimulus type across training condition by individual.

When retention test accuracy for each stimulus type is compared across training conditions, no pairwise comparison is significant, as shown in Table 2. The results are summarized in Figure 3, which shows mean centered accuracy for each stimulus type across the three training conditions. Plotting centered accuracy better represents data with a high degree of variation among individuals. Figure 4 illustrates the high degree of variation, with individual bar plots showing raw accuracy for each stimulus type across the three training conditions. The difference between the Control and two training groups was not significant (β = –0.19, SE = 0.23, z = –0.80, p = 0.42). This is likely due to a high degree of individual variation across all conditions, as indicated by the spread of retention accuracy within each group (Control: M = 0.63, SE = 0.035; Audiovisual Perception: M = 0.56, SE = 0.043; Audiovisual Production: M = 0.61, SE = 0.037). Overall, the results indicate that there is an effect of novel segments on retention accuracy but no effect of training.

### 3.2. Audiovisual Production Results

Within the Audiovisual Production group, the audio from the third repetition of each sound in training was extracted for qualitative rating by three raters. The three raters independently assigned a rating of 0–3 to each of the productions. A rating of 0 indicates that the participant produced the wrong sound consistently (e.g., [t^h^] instead of [ k͡| ]), and a rating of 3 indicates excellent and consistent production. Ratings were assessed for each of the four novel sounds (two novel consonants and two novel vowels), so each sound could have a total rating between 0 and 9, as a sum of the ratings given by the three raters, for each participant. Interrater reliability was assessed by calculating the intraclass correlation coefficient, using a two-way random-effects model compared against the mean rating for three raters (Koo and Li, 2016). The level of interrater reliability (r = 0.908, 95% CI [0.867, 0.938]) was classified as good to excellent. Participants’ scores were then averaged to indicate overall performance in the production practice session. This was intended to provide a broad measure of performance, but should not be interpreted on a sound-by-sound basis, especially as only novel sounds were scored.

A similar logistic mixed effects model was fit to the production data, with retention test accuracy as the dependent variable. Fixed effects included Production Score (continuous numerical score), and stimulus type (Familiar-Only, Novel-C, Novel-V, or Novel-CV) as well as their interaction. Production Score was found to significantly predict accuracy on the word-learning task (β = 0.53, SE = 0.21, z = 2.50, p = 0.012).

## 4. DISCUSSION

The results of this study support our initial hypothesis; existing pronunciation knowledge bootstraps word learning. Across all conditions, there is a trend in the data toward lower accuracy on items with two novel segments and higher accuracy on items with only familiar and novel consonant segments, shown in Figure 3. This is supported by statistical analysis in the comprehensive logistic mixed effects model, with significant or marginal results for the comparison of CV and Familiar in all conditions. While the effect size is relatively small, the consistency across categories indicates that words with two novel segments are indeed more difficult for learners to acquire. This finding fits with previous work, which has found that the presence of complex, unfamiliar consonant clusters negatively impacts word learning (Morrison and Hudson Kam, 2009).

The comparisons between accuracy for pseudowords with a single novel segment (C or V) are much less consistent. They do not show a statistically reliable pattern with respect to whether or not they reach, or come close to, significance. However, it is important to note that there is a trend consistent across conditions, with performance being the highest on Familiar or C, lowest on CV, and falling somewhere in the middle for V.

Broadly speaking, these results lead to two conclusions. First, novel vowels and novel consonants pattern differently. A possible explanation for the difference between the C and V conditions is the perceptual salience of the segments in question. Participants performed better on items with one novel consonant, which may have been more perceptually salient than those with the vowels, due to the comparatively crowded vowel space in English (Maddieson, 1984). This result, though unexpected, suggests that there may be an effect of segment type or salience on word learning. Second, while learners may be able to effectively learn and retain words with a single novel segment at more or less the same rate as words with only familiar segments, the same is not necessarily true when two novel segments occur together. This observation is consistent with the concept of superadditivity, in which the outcome exceeds the sum of the component parts. Superadditivity has been evoked in the multimodal sensory literature to account for how information from different senses, such as auditory and visual, combine and lead to greater intelligibility than the sum of their individual inputs (e.g., McGrath and Summerfield, 1985; Iverson et al., 1998). In this study, the inhibitory effect appears to be superadditive, with the presence of two novel segments leading to lower retention than the combined effect of the single-segment novel consonant and vowel lexical items. Future work should examine the role of pronunciation knowledge in more detail, specifically with respect to order, number, and sequence of segments.

This study entertained two competing hypotheses regarding the effect of production training on word learning. Given previous research, it was possible that introducing a pronunciation training paradigm could either help or hinder lexical acquisition. We found that there was no significant difference between the training conditions. This contradicts previous findings that production during training can cause an inhibitory effect (Baese-Berk and Samuel, 2016). However, these null results do not parallel the finding reported by Leach and Samuel (2007) either, where production provides a benefit when recognizing new words in noise. The differences from Baese-Berk and Samuel (2016) and Leach and Samuel (2007) may be due to the fact that this study used a single training session and a more passive methodology in the word-learning phase. However, this same explanation cannot be drawn in comparison with Morrison and Hudson Kam (2009), who found an inhibitory effect for production after a single training session.

It is interesting that while training did not lead to improved accuracy, neither did it hinder word learning. This provides motivation for further exploration, as two modalities of phonetic training were integrated and used in both experimental groups, namely ultrasound overlay videos and explicit articulatory instructions. Prior studies have largely used a more passive type of perceptual training, where participants listen to stimuli and make discrimination or categorization judgments. Little is known about the efficacy of using ultrasound videos for training (e.g., Bliss et al., 2017, 2018; Abel et al., 2015), and the results presented here indicate that further research is necessary to assess their effectiveness as a training method. The results here may also be due to the relative salience of the trained segments, which was purposefully emphasized in the present experimental design. Participants may be able to identify their mis-productions in ways they could not with perceptually challenging novel segments, especially in the case of the consonants. Performance on the lexical retention task was also associated with individual variability, observed across all experimental conditions. While we cannot draw comparisons with the Control or Audiovisual groups, the recordings captured during the training phase for the Audiovisual Production experimental group allow for some exploration of individual performance in production and word learning. In our analysis of the Audiovisual Production group, it was found that those who achieved a more accurate pronunciation overall in the training stage were more successful in the word-learning task. The Audiovisual Production results, reported in Section 3.2, suggest that participants who received higher production scores achieved higher accuracy in the word-learning task. While the role of production ability takes side stage to the primary questions of pronunciation knowledge and training type, the results offer insight into the questions, and provide fodder for future research.

The correlation between production performance in training and accuracy in lexical retention is indicative of individual differences among learners. We found that learners in the Audiovisual Production condition who achieved a more accurate pronunciation during training tended to perform better in the word-learning task. This supports an interpretation that individual differences, in part, contribute to word-learning performance. While prior research indicates many possible reasons for these differences, such as learner confidence and anxiety (Horwitz, 2001), we cannot speculate about such reasons in relation to the results of this study. Regardless, those who achieved a more native-like pronunciation of the L2 sounds performed better on the word retention task. Further research is necessary to assess why this might be the case; however, these results suggest that production ability does play a role in lexical acquisition, though the magnitude of this role may be mediated by complicated individual factors. It may have less to do with individual sounds and words, and more to do with learning ability. This is in line with the findings of Wong et al. (2007), who trained learners on English pseudowords with Mandarin tones. Using fMRI, they found activation patterns varied in participants before training and differences between more and less successful learners. This suggests a link between neural activation and language learning, where certain individuals seem to be more adept by virtue of physiological traits. While Wong et al. (2007) focused on learner perception of tone and word discrimination, it is plausible that these findings apply to production as well. Transfer between perception and production ability has been found in a number of L2 learning studies, such that perceptual success predicts production ability (Flege, 1993, 1999; Flege et al., 1997; Wang et al., 2003). In this study, if participants were better at perceiving the non-native sounds at the outset, this may have been reflected in their production. These speakers may been comparable to the successful participants in Wong et al. (2007), who approached the task with a more streamlined pattern of activation. As such, the Audiovisual Production results serve as a fruitful direction for future research, including work using fMRI methodology.

This study indicates that prior pronunciation knowledge is beneficial for word learning. While more work is needed to determine the shape and magnitude of this effect, the results offer direction; they suggest the importance of aspects such as segmental salience, number of novel segments, training design, and individual differences. The complicated role of production in word learning remains an important and promising avenue for future research in L2 acquisition.

## Figures and Tables

**Table 1.** Logistic mixed effects model *p*-values for accuracy by Stimulus Type, within Training Condition.

Training Condition		

Control	AV Perception	AV Production
C vs. Fam (*p* = 0.91)	C vs. Fam (*p* = 0.66)	C vs. Fam (*p* = 0.94)
V vs. Fam (*p* = 0.13)	V vs. Fam (*p* = 0.34)	V vs. Fam (*p* = 0.35)
^†^CV vs. Fam (*p* = 0.10)	^†^CV vs. Fam (*p* = 0.089)	*CV vs. Fam (*p* = 0.048)
^†^V vs. C (*p* = 0.092)	V vs. C (*p* = 0.59)	V vs. C (*p* = 0.28)
^†^CV vs. C (*p* = 0.063)	CV vs. C (*p* = 0.17)	*CV vs. C (*p* = 0.026)
CV vs. V (*p* = 0.87)	CV vs. V (*p* = 0.40)	CV vs. V (*p* = 0.23)

* Significance is assessed at *p* < 0.05.

† Values are considered marginal at *p* < 0.10.

**Table 2.** Logistic mixed effects model *p*-values for pairwise comparison of accuracy by Stimulus Type, across training conditions. Significance is assessed at *p* < 0.05.

Stimulus Type			

Familiar-Only	Novel-C	Novel-V	Nove-CV
Perception vs. Control (*p* = 0.43)	Perception vs. Control (*p* = 0.22)	Perception vs. Control (*p* = 0.57)	Perception vs. Control (*p* = 0.28)
Production vs. Control (*p* = 0.78)	Production vs. Control (*p* = 0.74)	Production vs. Control (*p* = 0.78)^5^	Production vs. Control (*p* = 0.59)
Perception vs. Production (*p* = 0.62)	Perception vs. Production (*p* = 0.38)	Perception vs. Production (*p* = 0.46)	Perception vs. Production (*p* = 0.60)

## Footnotes

1 We excluded participants enrolled in, or with credit for, LING 313 and LING 314 (or anything higher). These courses have substantial phonetic content, and we wanted participants with minimal linguistic background who were only enrolled in introductory courses.

2 Participants were excluded who reported fluency in the following languages, which contain at least one of the four novel sounds phonemically: Farsi, French, Greek, Japanese, Korean, Portuguese, Swedish, Thai, or Turkish. In the control group, there were nine L2 English speakers with Mandarin or Cantonese as L1 languages (mean age of L2 acquisition = 7.44 years) and one L1 bilingual speaker of Cantonese and English. In the Audiovisual Perception group, there were three L2 English speakers with Mandarin or Cantonese as L1 languages (mean age of L2 acquisition = 6 years) and one L1 bilingual speaker of Tagalog and English. In the Audiovisual Production group, there were eight L2 English speakers with Mandarin, Cantonese, Indonesian/Balinese, Vietnamese, or Punjabi as L1 languages (mean age of L2 acquisition = 6.13 years) and one Mandarin–Cantonese–English L1 trilingual. The rest of the speakers were L1 English speakers who did not report fluency in any other language.

3 Based on an internal survey of past studies, the most common native languages spoken by UBC Linguistics undergraduate students that participate in experimental studies are English, Mandarin, and Cantonese.

4 All participants reported that they speak English “fairly well” or “fluently.”

## APPENDIX

**Table:**

Stimuli			
Familiar-only	Novel-V	Novel-C	Novel-CV
fitu	føti	fɑχu	føχɑ
fɑɡu	føɡɑ	fɛχi	føk͡|i
fɛli	fɯlu	fuk͡|ɑ	fɯχu
fumɑ	fɯmi	fik͡|ɑ	fɯk͡|ɑ
ɡifɑ	ɡɯfu	ɡɑχu	ɡøχu
ɡɑtu	ɡøti	ɡuχi	ɡøk͡|ɑ
ɡɛlu	ɡølɑ	ɡɛk͡|i	ɡɯχi
ɡumɑ	ɡɯmɑ	ɡik͡|u	ɡɯk͡|i
lifu	lɯfɑ	luχi	løχi
luɡɑ	lɯɡu	luχɑ	løk͡|u
lɛtu	løti	lɑk͡|u	lɯχɑ
lɑmi	lømu	lɛk͡|u	lɯk͡|u
mifu	møfi	mɛχɑ	møχɑ
muɡɑ	mɯɡɑ	muχi	møk͡|u
muli	mɯlu	mik͡|u	mɯχi
mɑti	møti	muk͡|ɑ	mɯk͡|ɑ
